# Molecular identification of *Candida* species isolated from gastro-oesophageal candidiasis in Tehran, Iran 

**Published:** 2015

**Authors:** Rasoul Mohammadi, Saeed Abdi

**Affiliations:** 1 *Department of Medical Parasitology and Mycology, School of Medicine and Infectious Diseases and Tropical*; 2 *Medicine Research Center, Isfahan University of Medical Sciences, Isfahan, Iran*; 3 *Gastroenterology and Liver Diseases Research Center, Research Institute for Gastroenterology and Liver Diseases, Shahid Beheshti University of Medical Sciences, Tehran, Iran*

**Keywords:** *Candida *species, Gastro-oesophageal candidiasis, PCR-RFLP

## Abstract

**Aim:**

The aim of this investigation is identification of *Candida* strains isolated from patients with gastro-oesophageal candidiasis in Tehran, Iran.

**Background:**

Gastro-oesophageal candidiasis is a rare infection and appears mainly in debilitated or immunocompromised patients. Colonization by *Candida* spp. may occur in this region and the organism can remain for several months or years in the absence of inflammation. The main infection symptom is the presence of white plaques in gastro-oesophageal surface.* C. albicans *remains the most prevalent *Candida* spp. identified in gastrointestinal candidiasis. Regarding differences in susceptibilities to antifungal drugs among *Candida *spp., identification of isolates to the species level is significant to quick and appropriate therapy.

**Patients and methods:**

A total of 398 patients underwent gastrointestinal endoscopy during February 2012 to October 2014 were included in the present study. Histological sections from all endoscopic gastric and oesophageal biopsies were prepared, stained with Periodic acid–Schiff (PAS), and examined for the presence of fungal elements. Part of the biopsy sample was sub-cultured on sabouraud glucose agar. The genomic DNA of each strain was extracted using FTA^®^ Elute MicroCards. Molecular identification of *Candida* isolates was performed by PCR-RFLP technique with the restriction enzyme *Hpa*II.

**Results:**

Twenty-one out of 398 cases (5.2%) were found to have gastro-oesophageal candidiasis. *Candida albicans* was the main strain isolated from clinical samples (90.5%), followed by *C. glabrata* (4.7%), and *C. parapsilosis* (4.7%).

**Conclusion:**

Due to varying antifungal susceptibility of *Candida* spp. careful species designation for clinical isolates of *Candida* was recommended by a rapid and meticulous method like PCR-RFLP.

## Introduction

*Candida* species are the principal opportunistic yeast pathogens in human and most warm-blooded animals. Depending on the interaction between the fungal virulence factors, host defense mechanisms, and use of antifungal agents, colonization may be transient or tenacious and local disease may occur (1). After colonization, *Candida* spp. in low numbers may remain for several months or years in the absence of inflammation.* Candida *species are regularly isolated from the oral cavity and are found in 30%-60% of healthy individuals (2). Colonization rates were usually increased with the hospitalization period and acuteness of illness. Several factors increase the prevalence of esophageal and gastrointestinal (GI) candidiasis. The most prevalent reported cause of higher symptomatic oral and GI candidiasis is the antibiotics utilization specifically wide spectrum antibiotics like tetracycline (3). The esophagus is the second most frequent site of gastrointestinal candidiasis, after the oropharynx. The prevalence of *Candida *esophagitis (CE) has heightened mostly due to the association of infection with HIV-patients (4, 5). *Candida* species that are obtained from the esophageal surface are ordinarily the same organisms recognized in oral secretions (6). Gastro-oesophageal candidiasis is a rare infection and appears mainly in debilitated or immunocompromised patients. The infection symptom is the presence of white plaques in the gastro-oesophageal area confirmed by positive direct microscopic examination and cultures for the presence of *Candida* species (7). *C. albicans *remains the most prevalent *Candida* spp. identified in gastrointestinal candidiasis, including gastro-oesophageal region. Regarding differences in susceptibilities to antifungal drugs among *Candida *spp., identification of isolates at the species level is significant to quick and appropriate therapy. In this investigation, we present the *Candida* species distribution of clinical isolates obtained from gastro-oesophageal candidiasis using polymerase chain reaction-restriction fragment length polymorphisms (PCR-RFLP) method, to report the precise causative agent of gastro-oesophageal candidiasis. 

## Methods

A total of 398 patients underwent gastrointestinal endoscopy during February 2012 to October 2014 were included in the present study. Histological sections from all endoscopic gastric and oesophageal biopsies were prepared and stained with Periodic acid–Schiff (PAS), and examined for the presence of fungi. The criteria for the detection of *Candida* infection was the finding of infiltration by blastoconidia and pseudohyphae of tissue in histological sections of biopsies. A biopsy sample was taken, transferred into the serum saline, and sent to the laboratory for culture of microorganisms. The samples were subcultured on sabouraud glucose agar (Difco, Detroit, MI, USA) and CHROMagar Candida (CHROMagar Microbiology, Paris, France), and incubated at 32°C for 48-72 h. Genomic DNA of each strain was extracted using the FTA ® Elute MicroCards (Whatman Inc., Clifton, NJ, USA) according to the manufacturer’s instructions (8). Briefly, a loopful of a single colony was suspended in 80-100 μl of distilled water and 5 μl of the suspension was transferred to a disc of FTA card (4 mm in diameter) and incubated at 25°C for at least 5 h. The dried papers were eluted in 400 μl sterile water for 10 seconds, then the paper was transferred into a new micro-tube containing 40 μl distilled water and incubated at 95°C for 15 min. The paper discs were removed and the water, including DNA was used for PCR and stored at -20°C. Molecular identification of *Candida* strains was performed using delineated PCR-RFLP profiles (9, 10). Briefly, the ITS1-5.8SrDNA-ITS2 region was amplified using PCR mixture including 5μl of 10 × reaction buffer, 0.4 mM dNTPs, 1.5 mM MgCl2, 2.5 U of Taq polymerase, 30 pmol of both ITS1 (5′ -TCC GTA GGT GAA CCT GCG G-3′) and ITS4 (5′ -TCC TCC GCT TAT TGA TAT GC-3′) primers (11), as well as 2 μl of extracted DNA in a final volume of 50 μl. The PCR cycling conditions comprised: initial denaturation at 94°C for 5 min, followed by 30 cycles of denaturation at 94°C for 30 s, annealing at 55°C for 45 s, and extension at 72°C for 1 min, with a final extension at 72°C for 7 min. During the second step, PCR products were digested with the restriction enzyme *Hpa*II (Fermentas, Vilnius, Lithuania). Five microliters of each PCR amplicons and 10 μl of RFLP products were separated by gel electrophoresis on 1.5% and 2% agarose gel (containing 0.5 μg/ml ethidium bromide), respectively. 

**Table 1:** Characteristics of patient with Gastro-oesophageal candidiasis

No.	Gender	Age	White plaques on mucus surface	Predisposing factors	Clinical signs
Oesophageal candidiasis					
1	F	2 month	+	-	-
2	M	18	+	-	V
3	M	45	+	DM	EP
4	M	6 month	+	-	-
5	F	61	+	-	RP
6	M	22	-	-	EP
7	F	54	+	DM	V
8	F	36	+	-	H
9	F	67	+	DM	-
10	M	45	-	HD	D
11	F	19	+	-	V
12	M	4	+	HD	-
13	M	59	+	-	H
14	F	41	+	-	RP
15	M	29	+	HD	-
16	F	66	+	C	EP
17	F	51	+	-	V
18	M	37	+	UA	-
19	M	19	+	-	H
Oesophageal and gastric candidiasis					
20	M	60	-	DM	V
Gastric candidiasis					
21	M	66	+	-	EP+H

DM: Diabetes Mellitus, HD: Haematological disorders, C: Carcinoma, UA: Use of antibiotic, V: Vomiting, EP: Epigastric pain, RP: Retrosternal pain, H: Haematemesis, D: Dysphagia

## Results

**Figure 1:**
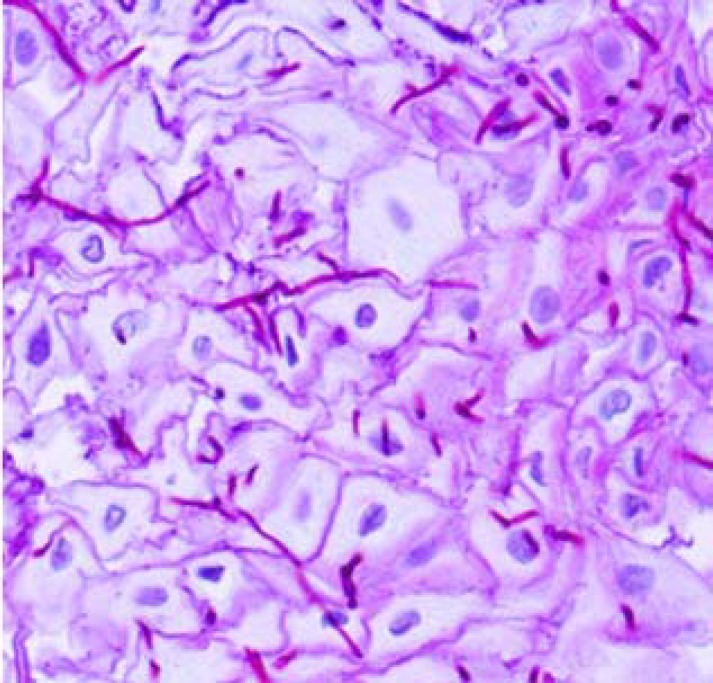
Fungal elements in oesophageal biopsies stained with Periodic acid–Schiff (PAS)

**Figure 2:**
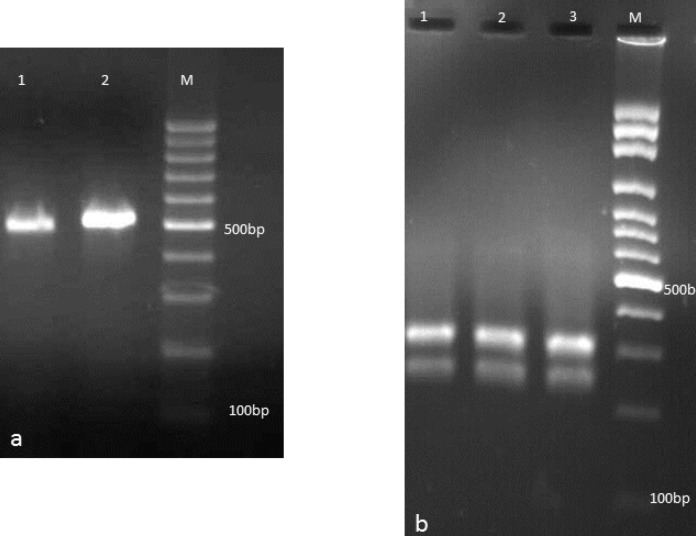
a: Agarose gel electrophoresis of ITS-PCR products of *Candida* isolates: Lanes 1,2 are *C. parapsilosis* and *C. albicans*, respectively. b: Agarose gel electrophoresis of ITS-PCR products of *Candida* isolates after digestion with *Hpa *II. Lanes 1-3 are *C. albicans*, and lanes M is 100 bp DNA size marker

Twenty-one patients were found to have gastro-oesophageal candidiasis with an incidence of 5.3% at endoscopy. Table 1 summarizes the details of patients included in the present study. Five patients (23.8%) were seen to have oral thrush plaque during the endoscopy. Three patients (14.3%) had hematological disorders, one patient (4.7%) had carcinoma, four patients (19%) were diabetic, and one patient (4.7%) took antibiotic for five days. According to endoscopic screening, white plaques were seen in all patients except for three patients. However, white patches were also seen in 8 out of 398 patients without histological signs. Two samples (9.5%) were obtained from gastric lesion, and 19 specimens (90.5%) were collected from esophageal ulcers. Histopathological findings (Figure 1) confirmed candidiasis in all patients except for two patients however, cultures of tissue biopsies were positive in all patients. *Candida albicans* was the main strain isolated from clinical samples (n=19, 90.5%), followed by *C. glabrata* (n=1, 4.7%), and *C. parapsilosis* (n=1, 4.7%) (Figure 2). Colony features on CHROM agar Candida confirmed our findings, as *C. albicans*, *C. parapsilosis*, and *C. glabrata* isolates gave distinctive green, white, and white colonies, respectively. Age range of patients was between two months and 67 years. Male to female ratio was 12/9. 

## Discussion

*Candida *spp. are the most prevalent causative agents of infectious esophagitis and, after the oropharynx, the esophagus is the second most common place of gastrointestinal candidiasis (1, 6). The prevalence of esophageal candidiasis has increased chiefly due to the immunosuppression diseases like AIDS. In agreement with the present study, many investigations show C*. albicans *as the most prevalent species identified in *Candida *esophagitis (1, 4, 5). *Candida *esophagitis in HIV-positive patients may be the first sign of the *Candida* infection, however, we didn’t have HIV-positive patient in the present study. The main esophageal candidiasis symptoms are odynophagia, dysphagia, epigastric pain, and retrosternal pain (1, 13). In the present study, four patients (19%) had epigastric pain, two patients (9.5%) had retrosternal pain, and one patient (4.7%) had dysphagia, however none of them had any odynophagia. A reliable diagnosis can only be made using direct imaging of the esophagus regions by endoscopy along with histological evidence of tissue invasion by *Candida* spp. in biopsy tissues (12). Histological evidence did not confirm candidiasis in two cases (9.5%) of this study. However, we can isolate *Candida* spp. in the culture of tissue biopsies in all patients. The differential diagnosis (DD) of *Candida *esophagitis must include viral esophagitis caused by herpes simplex virus or cytomegalovirus, idiopathic ulcers, and gastroesophageal reflux disease (13). However, histopathological findings and positive cell culture for *Candida* spp. in all cases changed our minds for gastro-oesophageal candidiasis. The incidence of gastric candidiasis in the present study (9.5%) was not in accordance with the incidence found by Scott and Jenkins (16%) (7), as well as Katzenstein and Maksem (18%) (14). Antifungal therapy using oral or parenteral fluconazole has been the cornerstone in the treatment of gastro-oesophageal candidiasis (4, 6). Topical antifungal agents like miconazole, clotrimazole, and nystatin are minimal values for the management of gastro-oesophageal candidiasis (15). Fluconazole revealed more rapid attack of symptoms (16). Patients treated with fluconazole (100-200 mg/day) had about 91% clinical response rates (17), close to the results of the present study (87%). Mimidis et al. (18) reviewed 55 patients diagnosed as *Candida* oesophagitis endoscopically and cytologically. Carcinoma, diabetes, steroids, gastric surgery and oesophageal motility disorders were considered as predisposing factors. They showed that twenty of 55 patients (36.3%) lacked the predisposing factor for* Candida* oesophagitis, whereas 11 out of 19 (57.9%) lacked any predisposing factor among patients with oesophageal candidiasis in the present study. Nishimura et al. (19) analyzed 733 HIV-infected patients who underwent upper gastrointestinal endoscopy. Of the 733 subjects, 62 (8.46%) were diagnosed with *Candidia* esophagitis. They revealed 55.2% (16/29) of the severe *Candidia* esophagitis patients had no GI signs and 44.4% (8/18) had no oral candidiasis. They reported 680 (92.8%) male patients while 57.1% patients were male in the present investigation.

Due to the varying antifungal susceptibility of *Candida* species and clinic-epidemiological reports, we emphasized on the careful species designation of the clinical isolates of *Candida* using PCR-RFLP as a rapid and meticulous method. 
